# *Cis*-Regulatory Timers for Developmental Gene Expression

**DOI:** 10.1371/journal.pbio.1001698

**Published:** 2013-10-29

**Authors:** Lionel Christiaen

**Affiliations:** Center for Developmental Genetics, Department of Biology, College of Arts and Sciences, New York University, New York, New York, United States of America

## Abstract

How does a fertilized egg decode its own genome to eventually develop into a mature animal? Each developing cell must activate a battery of genes in a timely manner and according to the function it will ultimately perform, but how? During development of the notochord—a structure akin to the vertebrate spine—in a simple marine invertebrate, an essential protein called Brachyury binds to specific sites in its target genes. A study just published in *PLOS Biology* reports that if the target gene contains multiple Brachyury-binding sites it will be activated early in development but if it contains only one site it will be activated later. Genes that contain no binding site can still be activated by Brachyury, but only indirectly by an earlier Brachyury-dependent gene product, so later than the directly activated genes. Thus, this study shows how several genes can interpret the presence of a single factor differently to become active at distinct times in development.

The development of a multicellular animal embryo from a single cell begins upon fertilization of the egg: the resulting zygote divides repeatedly to produce variable amounts of diverse early embryonic cells. These cells will go on to differentiate by activating distinct subsets of genes depending on their positions in the embryo and according to their prospective fates [1]. This cell-specific genome activation through transcription of specific genes involves nuclear proteins called transcription factors that bind to specific DNA sequences flanking the coding sequences of genes and called *cis*-regulatory modules (CRMs or enhancers). One or more specific transcription factors bind the CRMs in a target gene and then recruit and/or activate the basal transcription machinery to transcribe the coding sequence of the gene in a particular cell at a precise time [2],[3]. The interconnected activities of multiple transcription factors and their downstream target genes form tissue-specific gene regulatory networks (GRNs) [1]. GRN models are beginning to explain how genome sequence is converted into cell- and tissue-specific gene expression profiles [1],[4].

The marine invertebrate filter feeders called sea squirts, or ascidians, are among the closest relatives of the vertebrates. Various ascidian species such as *Ciona intestinalis* and *C.savignyi* provide simple model systems for chordate development: their larvae have the typical body-plan of animals with backbones, including a notochord—the precursor of the backbone. The ascidian notochord offers the opportunity to study, in a simple system, how the genomic blueprint directs the behavior of cells that form a specific structure during development. In 64-cell stage embryos, four cells start transcribing *Ci-Bra*, the gene that encodes the DNA-binding transcription factor Brachyury (Bra; Figure 1; [5],[6]). By the early gastrula stage, the four cells have divided once to form eight cells that maintain expression of *Ci-Bra* whereas two more cells from a different origin also activate *Ci-Bra* to form the secondary notochord lineage. These ten initial notochord precursors become internalized, divide twice, and rearrange during gastrulation to form an epithelium-like sheet of 40 post-mitotic cells. After gastrulation, in neurula and early tailbud embryos, where neurulation and early tail formation occur, cell intercalations cause the notochord to elongate by “convergent extension” to form a rod-shaped stack of cells [7],[8]. During the subsequent tailbud and larval stages, the rod-shaped stack of notochord cells elongate and begin to form the extracellular lumens that eventually fuse to create the tubular shape of the mature notochord [7],[8]. In *bra* mutants, notochord precursor cells are converted into endoderm and never undergo the characteristic notochord morphogenesis [9]. This indicates that Bra and its target genes are required for notochord cell behavior. Direct and indirect target genes of Bra are expressed sequentially during notochord development [10]–[12] and are required for specific notochord cell behaviors such as intercalation [7],[13],[14]. Yet Bra is expressed throughout and governs all aspects of early notochord development. So, how does it control the timing of essential effector genes that are deployed sequentially in successive morphogenetic phases?

**Figure 1:**
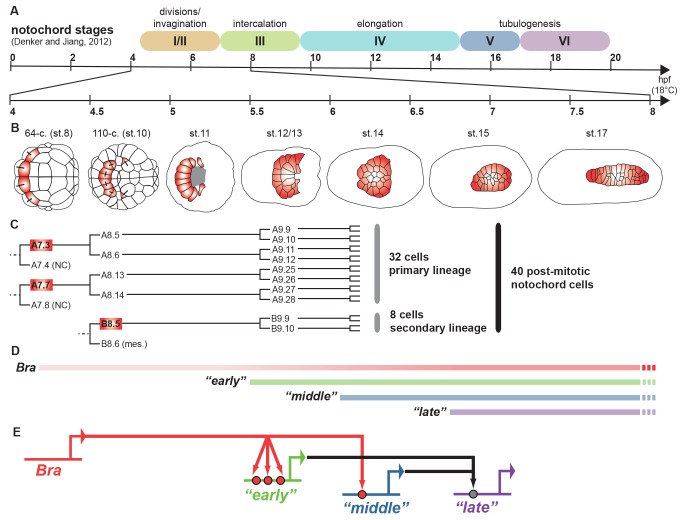
Early notochord development and temporal control of gene expression in *C. intestinalis*. (A) The stages and approximate timing of notochord development according to Denker and Jiang [7]. hpf, hours post fertilization. The approximate times corresponding to (B) through (E) are shown at higher magnification. (B) Schematic illustration of embryos at the indicated stages (after Hotta et al. [31]) showing the notochord cells (red). Bars connecting cells indicate sister cells following division. (C) Notochord cell lineages. Red boxes indicate the time of *Ci-Bra* expression. Only one side of the embryo is shown but cell numbers are for whole embryos. The cells are named following Conklin's nomenclature as in [5]. Note that cells expressing *Ci-Bra* arise from three separate lineages per side. Other tissue fates are shown as “NC” for nerve chord and “mes.” for mesenchyme. (D) Approximate timing of expression of the early-, middle, and late-onset genes regulated by Bra. (E) A simplified network showing early gene activation through three Bra-binding sites (red dots), middle gene activation through a single Bra site, and indirect late gene activation through an early and/or middle gene relay.

## A Simple *Cis*-Regulatory Code Controls Sequential Gene Activation

In a study reported in this issue of *PLOS Biology*, Anna Di Gregorio and colleagues analyzed the role of CRMs in the sequential activation of several Bra target genes in *C. intestinalis* [15]. They first focused on 11 of the numerous validated target genes and classified them as five “early,” four “middle,” and two “late” notochord genes according to their onset of expression. To identify notochord CRMs in these genes, the researchers transiently transfected embryos with fragments of the genome cloned into plasmid DNA containing a reporter gene to identify notochord-specific enhancer activity [5]. With this approach, they were able to isolate and characterize the minimal CRMs that specify early, middle, and late notochord gene expression.

In each of the early-onset notochord CRMs, the authors identified two to four copies of the consensus target sequence for Bra binding. By making mutations in the minimal CRMs, they found that only mutations of two or more sites abolished reporter gene expression, indicating that multiple functional and apparently redundant Bra-binding sites define the early-onset enhancer activity of notochord CRMs. By contrast, the enhancer activity of the middle-onset notochord CRMs relied on a single Bra-binding motif in each CRM.

The strict requirement for a single Bra site in middle-onset CRMs rather than the multiple sites in early-onset CRMs suggested that this might be the crucial factor that distinguishes between the early and middle onset of notochord gene expression in direct response to Bra. To test this possibility and determine precisely the developmental onset of transcription from early-onset and middle-onset CRMs, the authors recloned the CRMs upstream of their endogenous promoters to recapitulate the natural context of each CRM and analyzed the timing of reporter gene expression in the embryos. This analysis showed that, indeed, early-onset CRMs are active in 110-cell/early gastrula embryos, unlike middle-onset CRMs, whose enhancer activity is detected only at the late gastrula/neurula stage. Importantly, activation of the early-onset CRMs could be delayed to a middle-onset timing by mutating several of the Bra sites. This finding indicates that the multiple Bra sites in early-onset CRMs function synergistically and/or additively to permit gene activation at the early gastrula stage, even though individually they can direct later gene expression.

If the early- and middle-onset notochord genes are activated through multiple and single sites, respectively, how then are the late-onset genes activated? Analysis of a late-onset CRM identified binding sites for other putative transcription factors, such as homeodomain and Kruppel-like factors, but not for Bra. Consistent with this finding, chromatin immunoprecipitation (ChIP) assays showed direct binding of the endogenous Bra protein to early- and middle-onset CRMs but not to the late-onset CRM in tailbud embryos. Thus, it appears that Bra controls late-onset gene expression indirectly by a “relay” mechanism in which early- and/or middle-onset transcription regulators, such as those recently identified by the Di Gregorio team [16],[17], activate late-onset gene transcription (Figure 1).

Further investigation of the structural requirements for early-onset activity of notochord CRMs in response to Bra suggested that the observed synergistic effect between Bra-binding sites depends neither on the orientation, distance, or position of the functional sites relative to each other within the CRM, nor on a precise organization with respect to the promoter. Inspection of the non-coding sequences flanking orthologous notochord genes in the related species *C. savignyi* found only one early- and one middle-onset type CRM containing Bra-binding motifs and these were not systematically or perfectly conserved when compared to those in *C. intestinalis*. This further indicates that Bra-responsive CRM design is rather flexible and therefore variable even in evolutionarily related species, even though the underlying regulatory code may be conserved. Evolutionary conservation of the enhancer code was demonstrated previously in a comparative analysis of the CRMs controlling expression of the homeobox gene *Otx* from divergent ascidian species, where CRMs for neural, endodermal, and muscular expression relied on the same motifs (i.e., binding sites) in the two species even though the sequences of the CRMs could not be aligned [18]. Finally, the authors tested whether the presence of multiple versus single Bra sites could be used as a criterion to predict early- and middle-onset CRM activity. They found two novel CRMs, identified independently of this study, which controlled the early- and late-onset expression of notochord genes, as predicted on the basis of the number of Bra sites they contain.

## Diverse Modes of Temporal *Cis*-Regulation

How might multiple Bra-binding sites in a notochord CRM determine early onset of gene expression? One explanation is that the cluster of several sites might increase the overall affinity of the CRM for the transcription factor [19]. In this case, the CRM with the highest biochemical affinity will be activated by the lowest concentration of the transcription factor (the threshold–response model) [20]. A similar idea has been proposed to explain the temporal expression of the target genes of the transcription factor FoxA/PHA-4 during development of the *Caenorhabditis elegans* pharynx, where individual sites of high binding affinity for PHA-4 determine early onset of target gene expression [21]. Di Gregorio and colleagues did not report different affinities between the Bra sites they found in early- versus middle-onset CRMs, however, suggesting that differences in site numbers may be the main determinants of different CRM affinities for Bra.

This threshold–response model also implies that the availability of Bra protein is limiting for activation of middle-onset CRMs in early gastrula versus early neurula embryos. The availability of Bra might be determined by its gene expression, control of its accumulation in the nucleus, how its target DNA is packaged in chromatin, and/or competition with other DNA-binding proteins (for example, transcription repressors) [2],[3].

The threshold–response model of gene activation has been extensively tested during early development of the fruitfly, *Drosophila melanogaster*, where the transcription factor Dorsal controls dorso-ventral patterning. Like the Bra target genes, the sensitive Dorsal target gene *sog*, which is governed by a multi-site CRM [19], is activated earlier than the less-sensitive targets *sna* and *vnd* [21]. Imaging of a fluorescent Dorsal-YFP fusion protein in living embryos showed that the concentration of Dorsal in the ventral nuclei, where it is the highest at any given time, increases between and during cell cycles [21]. A similar increase in Bra protein concentration over time would be consistent with the observed steady rate of mRNA transcription [7] and might contribute to the sequential activation of early- and middle-onset notochord gene expression, which may also be separated by cell divisions (Figure 1B and 1C), as is the case in the early fly embryo [22]. In future studies, it would be interesting to address whether the nuclear concentration of Bra increases over time, within and/or in between the cell cycles following *bra* activation, and whether the temporally distinct onsets of notochord gene expression coincide with successive divisions.

Studies in *Drosophila* indicate that transcription factor binding is not necessarily sufficient for transcriptional activation: some sites bind their transcription factors early but activate transcription only later when other factors have bound [22]. The binding of the transcription factor biniou (bin) during *Drosophila* visceral mesoderm development illustrates this idea [23]. This study identified distinct classes of CRMs according to the time at which they bound bin: the classes that bound bin early contained binding motifs for various combinations of *trans*-acting factors, whereas the class that bound bin late contained only bin-binding sequences, suggesting that early bin binding requires cooperation with other DNA-binding factors. Comprehensive ChIP and expression studies further substantiated the notion that combinatorial transcription factor binding determines the spatio-temporal pattern of CRM activity in the fly mesoderm [24].

The control of ascidian notochord development by Bra might be simpler than that of the *Drosophila* ventral mesoderm, however. Whereas the *Drosophila* mesoderm is a complex population of transient progenitors with diverse developmental outcomes and proliferative potential, the ascidian notochord cells are fate-restricted at the onset of *Ci-Bra* expression and become post-mitotic after only two divisions. In other words, ascidian notochord cells undergo terminal differentiation following *bra* expression. As in *C. elegans* post-mitotic neurons and male tail tip differentiation [25],[26], it is possible that a single terminal selector gene, *Ci-Bra*, controls most aspects of notochord differentiation in ascidians. However, additional transcription regulators, such as FoxA and Tbx2/3, govern late notochord gene expression and probably also contribute to regulating early- and middle-onset gene expression [16],[17],[27]. These additional transcription regulators, acting downstream of and/or in parallel to Bra, suggest that coherent feed-forward loops, whereby Bra activates a downstream factor that then cooperates with Bra to activate further downstream targets, and maybe also other gene regulatory network motifs [28], control the onset of notochord gene expression.

## Towards a Systems Level Understanding of Morphogenesis

The premise that timely activation of notochord genes is essential for the morphogenesis of this structure remains to be tested functionally. Recent work on the early *Drosophila* embryo showed that asynchronous expression of the early determinant *sna* throughout the mesoderm profoundly affects gastrulation [29]. In the early fly mesoderm, synchronous gene expression among nuclei within a given gene expression domain appears to be mediated by RNA polymerase II pausing, which depends on the activity of the promoter rather than the CRM [29]. This transcriptional pausing phenomenon together with an averaging of transcript abundance over time may buffer the naturally stochastic bursts of transcription [30]. Thus, understanding the role of sequential gene transcription in developmental biology will require not only the ability to determine the quantitative influence of regulatory sequences on transcription factor binding and CRM activity, but also the ability to measure the actual transcriptional responses by using creative molecular tools, quantitative imaging methods, and computational modeling. Finally, a complete understanding of notochord morphogenesis will be reached only when these transcriptional responses will be used to explain quantitatively the timing of the sub-cellular biochemistry and biophysics underlying the phenomenon.
